# Current News and Opinions

**Published:** 1889-01

**Authors:** 


### Current News.

Who will play the *Review* organ?

Dr. Alton Thompson has resigned the editorial chair. We are sorry to lose Dr. T. from our ranks.

Dr. A. Morseman has accepted the position of associate editor made vacant by the resignation of Dr. Thompson. We congratulate the *Western Dental Journal* on its acquisition.

H. D. Justi has undertaken the publication of the *Dental Review*. The editors claim that the journal has not departed from its position as an independent journal, but the publishers say that "they have an organ now."

Dr. J. D. Patterson, the genial editor of the *Western Dental Journal*, made a hurried call upon us in our sanctum the past week. He was anxiously enquiring for the last issue of his journal. We appreciate your feelings, Doctor. We have been away from home too, and wondered whether things would go all right in our absence

### THE TWENTY-FIFTH ANNIVERSARY OF THE CHICAGO DENTAL SOCIETY

Will be celebrated by a Three Days' Meeting to be held in the Ladies' Ordinary, of the Grand Pacific Hotel, corner of Clark and Jackson Streets, Chicago, Ill., February 5, 6, and 7, 1889.

The Grand Pacific Hotel will be the headquarters for guests, and will furnish rooms above the parlor floor, with board, at three dollars per day. All other rooms at fifty cents per day less than usual rates.

The committee expect to secure reduced rates, therefore the *usual receipts should be taken* when railroad tickets are purchased showing the payment of full fare, so as to secure reduced rates returning where it is possible.

An exhibit will be made by manufacturers and dealers of novelties in their various lines.

The meetings will be exclusively devoted to the reading of papers and the discussion of professional subjects, and no other business will be transacted.

PROGRAMME. TUESDAY MORNING, FEB. 5, 1889.

The meeting will be called to order promptly at 10 o'clock.

PRAYER

By Rev. G. C. Lorimer, D.D., of Chicago.

PAPER—" Gum-colored Porcelain Fillings," by A. H. Thompson, Topeka, Kansas.

DISCUSSION.

PAPER—" A Study of the Effects of Cocaine upon Man and Some of the Lower Animals," by C. P. Pruyn, Chicago, Illinois.

PAPER—" Obtundents of Sensitive Dentine," by T. E. Weeks, Minneapolis, Minn.

TUESDAY EVENING, 7.30 O'CLOCK.

PAPER—" The Study of Pre-historic Remains in their Relation to Dentistry," by J. J. R. Patrick, Belleville, Illinois.

DISCUSSION.

PAPER—" Caries and Necrosis in the Relation to Practical Dentistry," by J. H. Martindale, Minneapolis, Minn.

WEDNESDAY MORNING, 9 O'CLOCK.

CLINICS at the Chicago College of Dental Surgery.

WEDNESDAY AFTERNOON, 3 O'CLOCK.

PAPER—" Antiseptics," by G. V. Black, Chicago, Illinois.

WEDNESDAY EVENING, 7.30 O'CLOCK.

PAPER, with Lantern Illustrations—" The Development of the Teeth, the Formation of Dentine, and its Appearance in Health and Decay." The paper will be illustrated by photo-micrographs, projected on the screen by means of the oxy-hydrogen lantern. Many of the photographs were made for this demonstration; others are from Dr. W. D. Miller's beautiful specimens of Natural and Artificial Decay. By R. R. Andrews, Cambridge, Mass.

DISCUSSION.

PAPER—" Artistic Methods in Prosthetic Dentistry," by L. W. Comstock, Indianapolis, Ind. Illustrated by large cartoons.

CLINICS will be held on Wednesday and Thursday mornings at the Chicago College of Dental Surgery, northeast corner Wabash Avenue and Madison Street. They will begin promptly at 9 o'clock.

WEDNESDAY, FEB. 6.

J. B. Vernon, St. Louis, Mo.; Bridge Work.

C. Thomas, Des Moines, Iowa; Porcelain Fillings.

Francis Peabody, Louisville, Ky.; Filling Root Canals with Lead Points.

C. S. Case, Jackson, Mich., will demonstrate his method of making artificial vela and obturators for cleft palate, provided a subject can be secured.

A. H. Thompson, Topeka, Kansas; Gum-colored Porcelain Fillings.

E. T. Darby, Philadelphia, Pa.; Filling with Crystal Gold; and the use of Matrices.

A. W. Hoyt, Chicago, Ill.; Porcelain Fillings secured by Gold Filling.

S. G. Perry, New York City, will demonstrate the application of Perry's Separators, and the Weber-Perry Engine and Mallet.

T. E. Weeks, Minneapolis, Minn.; Setting of Logan Crown with Gold Attachment, showing original method of Investment for Soldering.

D. F. Mc Graw, Mankats, Minn.; Obtunding of Sensitive Dentine, and Controlling of Peri-Dental Inflammation by Electrolysis.

J. W. Wick, St. Louis, Mo.; His method of Gold Filling.

Louis Ottofy, Chicago, Ill.; Implantation.

THURSDAY, FEB. 7.

T. D. Gilmer, Quincy, Ill.; Gold Crown Telescoped over a Platinum Band; also a Combination Crown of Platinum and Weston's Metal, or of Gold, Porcelain, and Weston's Metal.

E. H. Allen, Freeport, Ill.; Gold Filling, using Electric Mallet.

C. N. Johnson, Chicago, Ill.; Gold Filling.

C. W. Lewis, Chicago, Ill.; Herbst Method.

J. G. Reid, Chicago, Ill.; Copper Amalgam.

M. E. Smith, Chicago, Ill.; Gold Filling, using Snow & Lewis Plugger.

W. H. Taggart, Freeport, Ill., will show a new Root Trimmer, and a new Suspension Engine.

J. W. Wassall, Chicago, Ill., will demonstrate Root Filling with Chlora-percha and Gold Points; also the use of McKellop's Platinum Gold Broaches.

E. A. Royce, Chicago, Ill.; Gold Filling, using Abbey's Non-Cohesive Gold in Cylinders.

T. S. Waters, Baltimore, Md.; Movable Bridge.

R. B. Winder, Baltimore, Md.; Movable Bridge; also a new Rubber Dam Clamp, combined with a Cheek Holder; also a separator.

Henry A. Parr, New York City; Movable Bridge.

J. A. Woodward, Philadelphia, Pa.; Reflector for lighting the mouth.

J. N. Farrar, New York City, is expected to be here, and will exhibit his Regulating Appliances.

J. N. CROUSE,<br>

GEO. H. CUSHING,<br>

E. NOYES, Executive Committee.

